# Periodic Membrane Potential and Ca^2+^ Oscillations in T Cells Forming an Immune Synapse

**DOI:** 10.3390/ijms21051568

**Published:** 2020-02-25

**Authors:** Ferenc Papp, Peter Hajdu, Gabor Tajti, Agnes Toth, Eva Nagy, Zsolt Fazekas, Sandor Kovacs, György Vámosi, Zoltan Varga, Gyorgy Panyi

**Affiliations:** 1 Department of Biophysics and Cell Biology, Faculty of Medicine, University of Debrecen, H-4032 Debrecen, Hungary; papp.ferenc@med.unideb.hu (F.P.); tajti.gabor@med.unideb.hu (G.T.); agi.toth@yahoo.com (A.T.); even@mailbox.hu (E.N.); fzsolt@med.unideb.hu (Z.F.); vamosig@med.unideb.hu (G.V.); veze@med.unideb.hu (Z.V.); 2 Department of Biophysics and Cell Biology, Faculty of Dentistry, University of Debrecen, H-4032 Debrecen, Hungary; hajdup@med.unideb.hu; 3 Institute of Sectoral Economics and Methodology, Faculty of Economics and Business, Department of Statistics and Research Methodology, University of Debrecen, 4032 Debrecen, Hungary; kovacs.sandor@econ.unideb.hu

**Keywords:** T-cell, membrane potential, oscillation, immunological synapse, Kv1.3, KCa3.1, CRAC

## Abstract

The immunological synapse (IS) is a specialized contact area formed between a T cell and an antigen presenting cell (APC). Besides molecules directly involved in antigen recognition such as the TCR/CD3 complex, ion channels important in the membrane potential and intracellular free Ca^2+^ concentration control of T cells are also recruited into the IS. These are the voltage-gated Kv1.3 and Ca^2+^-activated KCa3.1 K^+^ channels and the calcium release-activated Ca^2+^ channel (CRAC). However, the consequence of this recruitment on membrane potential and Ca^2+^ level control is not known. Here we demonstrate that the membrane potential (MP) of murine T cells conjugated with APCs in an IS shows characteristic oscillations. We found that depolarization of the membrane by current injection or by increased extracellular K^+^ concentration produced membrane potential oscillations (MPO) significantly more frequently in conjugated T cells than in lone T cells. Furthermore, oscillation of the free intracellular Ca^2+^ concentration could also be observed more frequently in cells forming an IS than in lone cells. We suggest that in the IS the special arrangement of channels and the constrained space between the interacting cells creates a favorable environment for these oscillations, which may enhance the signaling process leading to T cell activation.

## 1. Introduction

The role of the membrane potential and voltage-gated K^+^ (Kv) channels in the activation of T lymphocytes was established more than three decades ago [1]. Depolarization of the cells by elevated external K^+^ concentration or by blocking the Kv channels inhibited T cell mitogenesis [2,3,4]. Soon after, the relevance of the changing cytosolic Ca^2+^ concentration or Ca^2+^ signaling in the activation pathway has also become clear. Some studies have reported periodic Ca^2+^ oscillations, which were suggested to be required for activation [5,6]. Membrane potential fluctuations have also been detected in T cells and their connection to the periodic Ca^2+^ signals has been investigated [7,8,9].

Via positive and negative feedback loops the concerted interplay of ion channels generates dynamic changes in the membrane potential and ionic fluxes [10]. In resting T cells the voltage-gated Kv1.3 K^+^ channel is mostly responsible for setting the membrane potential at about -50 mV [9]. Its steep sigmoid voltage-dependence establishes a negative feedback loop, as membrane depolarization opens the channel and the consequent K^+^ efflux hyperpolarizes the membrane leading to channel closure [11,12,13,14]. Another K^+^ channel, present in much lower numbers in resting naïve T cells, but at substantially elevated numbers in activated T cells, is the Ca^2+^-activated K^+^ channel KCa3.1 [15]. It is activated by the rise of the cytosolic Ca^2+^ concentration and its opening causes K^+^ efflux and therefore hyperpolarization similarly to Kv1.3. A third important player in this channel network is the CRAC (Ca^2+^-release activated Ca^2+^) channel, which consists of the Ca^2+^ sensor STIM1 in the endoplasmic reticulum (ER) and the pore-forming subunit Orai1 in the plasma membrane [16,17]. When the TCR/CD3 complex binds the presented antigen, the downstream signaling events lead to IP_3_ production with a consequent release of Ca^2+^ from the ER and activation of CRAC channels. The extremely low conductance CRAC channels [18] are activated by emptying of the ER Ca^2+^ store sensed by STIM1 [19] and allow Ca^2+^ influx. Ca^2+^ influx enhances the activity of Kv1.3 by causing depolarization and that of KCa3.1 by elevating the cytosolic Ca^2+^ concentration. In turn, K^+^ efflux through these channels helps to maintain the driving force for the Ca^2+^ influx. Additional channels, such as members of the two-pore-domain K^+^ channel family have been suggested to be important in controlling the Ca^2+^ signaling of T cells [20], but most experimental evidence supports the dominant role of the above mentioned three channels.

Although these regulatory principles apply in general, the actual course of membrane potential and cytosolic Ca^2+^ concentration changes can be profoundly influenced by the relative number of the involved channels, their respective distribution in the membrane and possible geometrical constraints, such as enclosed areas, where diffusion of even small ions is limited. Various signals may induce reorganization of the channel distribution and co-localization, and the up- or down-regulation of certain channel types. In these scenarios local effects such as accumulation or depletion of ions in the immediate vicinity of a channel or local electric potential changes may produce phenomena not normally present under usual conditions. Such circumstances may arise during the formation of an immunological synapse (IS) formed between an antigen-presenting cell (APC) and an antigen recognizing T cell. The IS is a signaling platform where molecules involved in antigen recognition and subsequent signal transduction events are dynamically recruited upon conjugation of the interacting cells [21,22]. In addition to molecules directly involved in antigen recognition such as the T cell receptor/CD3 complex and associated kinases [23], the above mentioned ion channels are also translocated into this signaling platform of T cells [13,14,15,24]. Earlier we and others have shown that Kv1.3 [11,12,13,14], KCa3.1 [15] as well as the calcium release-activated Ca^2+^ channels (CRAC) [24,25,26] are recruited into the IS. Although the consequences of their localization in the IS are not clear [27,28], the high channel densities make strong interactions possible. The “synaptic cleft” between the cells represents an enclosed space with high molecular crowding, possibly creating ionic concentrations that significantly differ from those in the bulk solution. Theoretical calculations show that significant accumulation of K^+^ ions and depletion of Ca^2+^ ions is possible in this restricted volume [10,29].

We set out to investigate if these special conditions could generate special membrane potential changes or patterns in the intracellular Ca^2+^ concentration by comparing membrane potential and free Ca^2+^ concentration recordings from lone T cells and T cells forming immunological synapses. We have found that the membrane potential of murine T cells conjugated with APCs in an IS shows characteristic oscillations unlike the fluctuations described earlier. We found that depolarization of the membrane by injection of a +5 to +20 pA current into conjugated T cells or by increasing the extracellular K^+^ concentration produced membrane potential oscillations (MPO) significantly more frequently in conjugated T cells than in cells alone. Charybdotoxin (blocker of both Kv1.3 and KCa3.1 channels) depolarized the membrane potential and decreased both the frequency and the amplitude of the MPO. TRAM-34, a selective blocker of KCa3.1 channels, only reduced the amplitude of the MPO. In addition, oscillation of the free intracellular Ca^2+^ concentration could also be observed more frequently in cells forming an IS than in lone cells.

Although membrane potential fluctuations have already been described in T cells, they lacked the periodicity and amplitude of the oscillations shown here [7,8,9]. We suggest that the special conditions created by the IS formation facilitate the generation of these characteristic MPOs along with the accompanying Ca^2+^ oscillations, which may modulate the signaling cascade of T cell activation.

## 2. Results

### 2.1. Characterization of Kv1.3 and KCa3.1 Ion Channels Expressed in D10 T Cells and Formation of an Immune Synapse with CH12 B Cells

A murine model system was used to address our hypothesis, where CH12 B cells present conalbumin antigens to D10 murine helper T cells specific for this antigen [22]. Whole-cell patch-clamp was used to characterize the ion channel expression of D10 cells, which showed that K^+^ channels were expressed on these cells.

**Figure 1:**
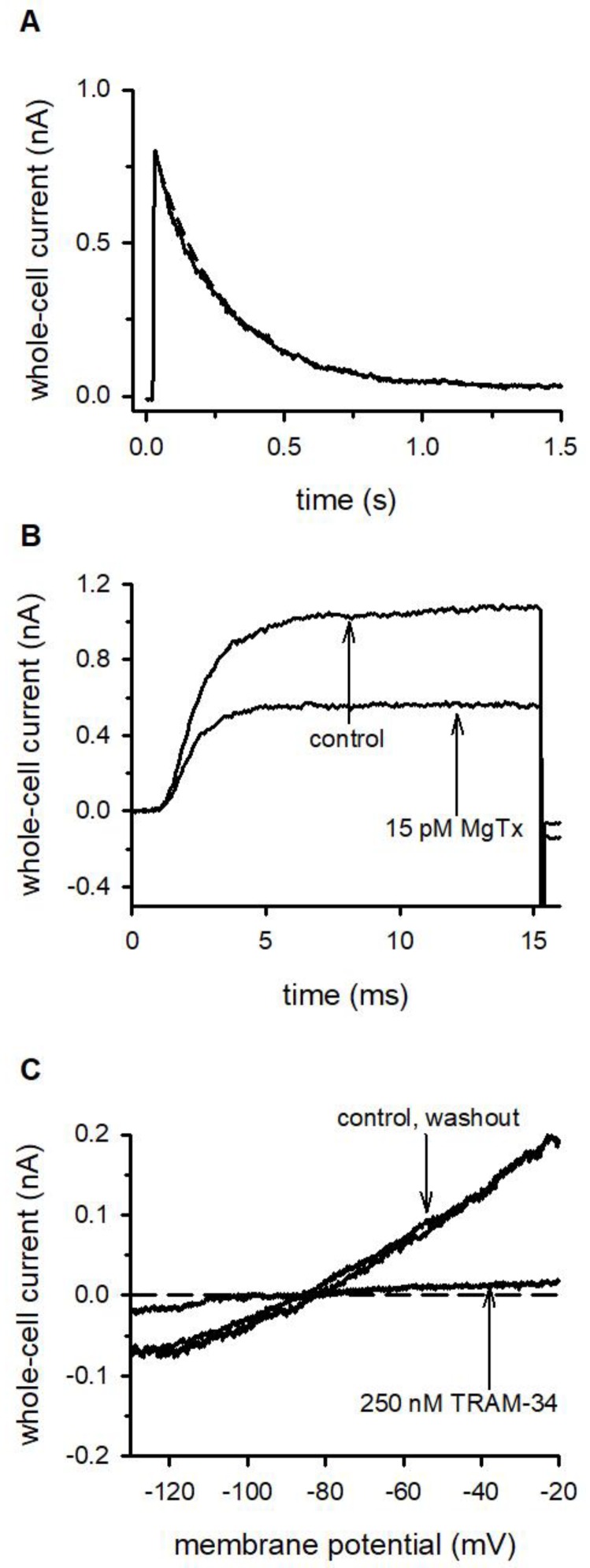
Kv1.3 and KCa3.1 currents are expressed in D10 cells. (**A**): Representative Kv1.3 K^+^ current in a single D10 cell recorded during a 1.5-s-long test pulse to +50 mV from a holding potential of –120 mV. The superimposed dashed line indicates the best fit single exponential with τ =252 ms. (**B**): Representative Kv1.3 K^+^ currents from a single D10 cell in control solution, and after the equilibration of the block in the presence of 15 pM MgTx (test pulse: +50 mV). (**C**): Voltage ramps from –120 mV to +50 mV (duration: 150 ms) evoked KCa3.1 currents from a single D10 cell. Traces show the current in control solution, after the equilibration of the block in the presence of 250 nM TRAM-34, and after wash-out. The voltage range below the activation threshold of Kv1.3 channels is shown only.

**Figure 2:**
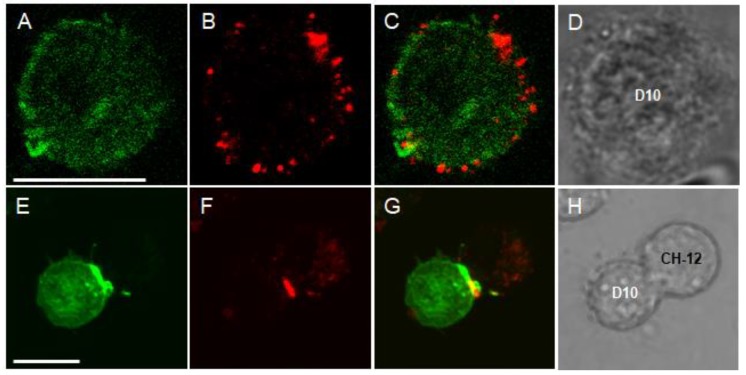
Recruitment of PKCθ-GFP and Kv1.3 into the IS. Representative confocal images of a D10 cell alone (**A**–**D**) or conjugated to a CH12 cell (E-H). Panels from left to right display: (**A**,**E**): GFP signal of PKCΘ (green), (**B**,**F**): Cy3 fluorescence of Kv1.3 signal (red), (**C**,**G**): merge of the PKCθ-GFP and Kv1.3 signals. (**D**,**H**): bright field image of the cells. Slice thickness was set to 1 μm. The image was taken 20 min after mixing and centrifuging together the two cell types. Scale bar is 10 µm.

**Figure A1:**
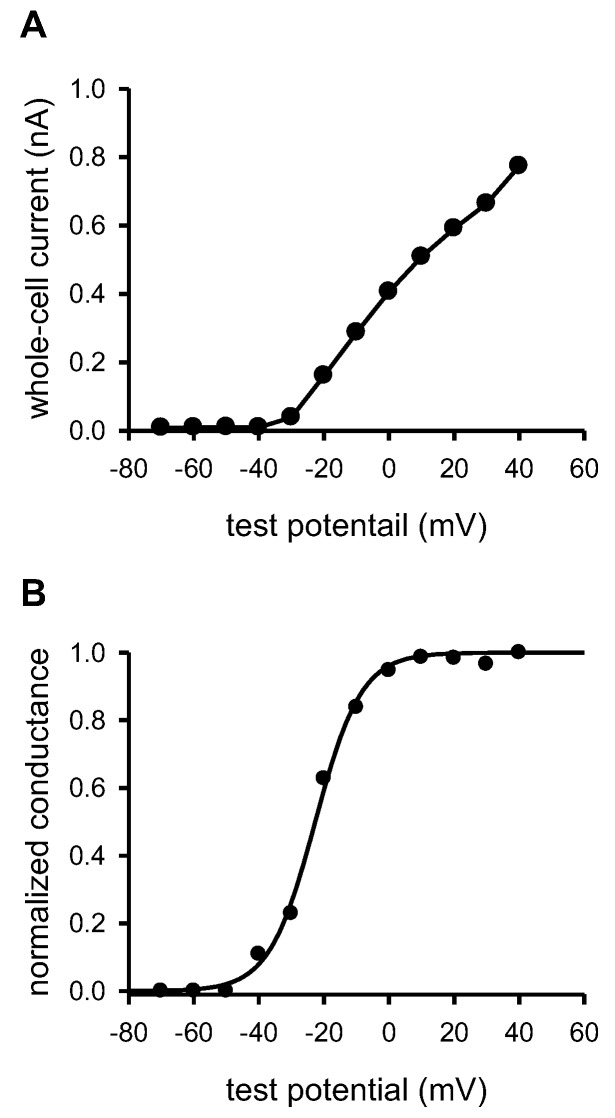
Depolarization-activated, outward potassium current in D10 cells. (**A**): The current-voltage relationship for the Kv1.3 K+ current was determined using a standard protocol: cells were held at −120 mV and depolarized to the test potentials between –70 mV to +40 mV in 10 mV steps in every 30 s. The whole-cell peak currents detected at the indicated test potentials were plotted versus the test potential. (**B**): Voltage-dependence of steady-state activation for the same cell as in panel A. Peak whole-cell conductance (G(V)) at each test potential was calculated from the peak current (Ip) at test potential V and the K+ reversal potential (Er) using G(V)=Ip/(V-Er). G(V) values were normalized for maximum conductance and plotted as a function of test potential along with the best fit Boltzmann-function (normalized conductance (GN) = 1/(1+exp [-(V-V½)/k]). For this cell the fitting gave −22 mV and 7.1 mV for V½, and k, respectively.

Figure 1A shows that voltage-clamped D10 cells display a quickly activating and slowly inactivating current when the membrane potential is depolarized to +50 mV from a holding potential of −120 mV. Regarding its voltage dependence and kinetic parameters this whole-cell current is similar to the one recorded in human peripheral blood T cells expressing Kv1.3 channels [3,30]. The average current amplitude at +50 mV was 441 ± 89 pA (*n* = 18). The expression of Kv1.3 channels in D10 cells was confirmed by the inhibition of the current by margatoxin (MgTx), a high affinity inhibitor of Kv1.3 channels applied at its known blocking concentration [31] (Figure 1B), determining the midpoint of the voltage dependence of steady-state activation (V_1/2_ = −25 ± 2 mV, *n* = 13, Figure A1, panel B) and the time constant of inactivation kinetics (τ = 364 ± 26 ms, *n* = 17, Figure 1A). A pipette solution having 1 μM free Ca^2+^ concentration was used to measure the expression of KCa3.1 channels in D10 cells in response to voltage ramps from −120 mV to +50 mV [32]. The slope of the current below the activation threshold of Kv1.3 is characteristic for the KCa3.1 conductance of the membrane. The current magnitude was 209 ± 33 pA at −20 mV membrane potential (*n* = 12). The presence of KCa3.1 channels was confirmed by the inhibition of the whole–cell current by TRAM-34 [33], a selective small molecule inhibitor of these channels (Figure 1C). The formation of cell conjugates between APCs and T cells was initiated by co-centrifugation of conalbumin antigen-pulsed CH12 cells and D10 cells at a ratio of 1:1 [22]. The formation of the IS was confirmed by the characteristic recruitment of the GFP-tagged PKCΘ into the synapse using confocal microscopy (Figure 2) [22,34]. In subsequent experiments the specific recruitment of PKCΘ-GFP into the IS was used to identify suitable cell conjugates for electrophysiological experiments.

### 2.2. The Membrane Potential Oscillates More Frequently in Conjugated T Cells Than in Lone T Cells

**Figure 3:**
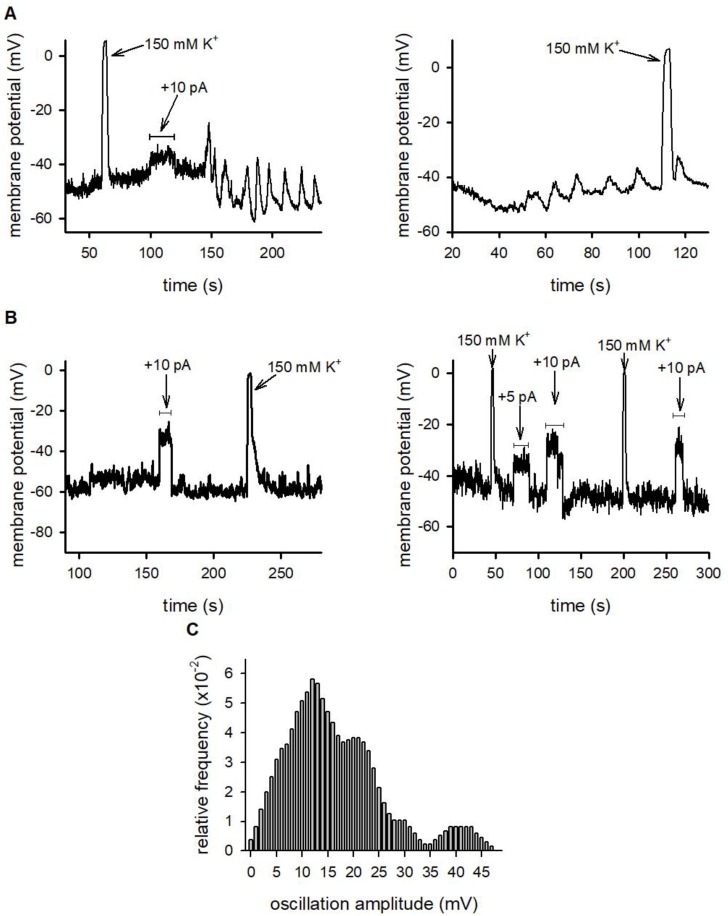
Membrane potential responses of IS conjugated and lone T cells. (**A**): D10 T cells engaged in IS with CH12 cells. The membrane potential was measured in the current-clamp mode. Left panel: the horizontal line indicates the duration (20 s) of the depolarizing current injection (+10 pA) followed by MPO in a D10 cell (representative trace). Right panel: the MPO oscillates spontaneously in a D10 cell (representative trace). When indicated (arrows) the recording chamber was perfused with 150 mM K^+^ extracellular solution. (**B**): lone D10 cells (not engaged in IS). Left and right: representative membrane potential records (current-clamp) of two D10 cells. Horizontal lines indicate the durations of the depolarizing current injections (+ 5 pA or +10 pA, as indicated). When indicated (arrows) the recording chamber was perfused with 150 mM K^+^ extracellular solution. (**C**): Distribution of the amplitudes of membrane potential oscillations measured on D10 T cells engaged in IS. Smooth histograms were created by an averaging procedure as described in the Methods.

We recorded the membrane potential of D10 cells not conjugated (“lone”) or conjugated with specific antigen presenting CH12 cells using the patch-clamp technique [35] in I = 0 current clamp mode. As leak resistance dramatically interferes with membrane potential determination, we included only those records in the analysis where the seal resistance was greater than 1 GΩ. The instantaneous, reversible depolarization of the resting membrane potential to ~0 mV in the presence of 150 mM extracellular K^+^ was used as an indicator of the reliability of membrane potential determinations (Figure 3). The resting potential of lone and conjugated D10 cells were −51.8 ± 4.7 mV (*n* = 18) and −52.7 ± 3.1 mV (*n* = 25), respectively, in good agreement with the literature [8]. During antigen presentation the opening of CRAC channels and the concomitant Ca^2+^ influx produces an inward cation current [36]. Similar Ca^2+^ currents up to about 10 pA amplitude were induced by thapsigargin or PHA stimulation in Jukat T cells [19]. The depolarizing effect of this current was mimicked in our experiments by applying depolarizing current pulses to current clamped D10 cells with amplitudes of +5 to +20 pA (durations indicated in Figure 3). In the majority of the D10 cells engaged in an IS with CH12 B cells (13 out of 22 cells, 59%) we detected high amplitude periodic membrane potential oscillations in response to such current injections (Figure 3A, left) or to depolarization by 150 mM external K^+^. In some cells oscillation developed spontaneously (Figure 3A, right). In contrast, a much smaller fraction (four out of 18 cells, 22%) of lone cells produced such oscillations under similar conditions, most were unresponsive (Figure 3B, left and right panels). The ratio of the occurrence of oscillations was significantly higher in conjugated cells than in lone cells indicating that the presence of MPO is not independent from the immunological synapse formation (Fisher’s exact test, *p* = 0.027). Membrane potential oscillations were transient: MPOs induced by either method lasted for ~50 to 300 s and ceased spontaneously.

In order to make an objective decision about the presence or absence of MPO we developed a mathematical method analysing the distribution of the data points in the membrane potential recordings (see details in Materials and Methods).

**Figure 4:**
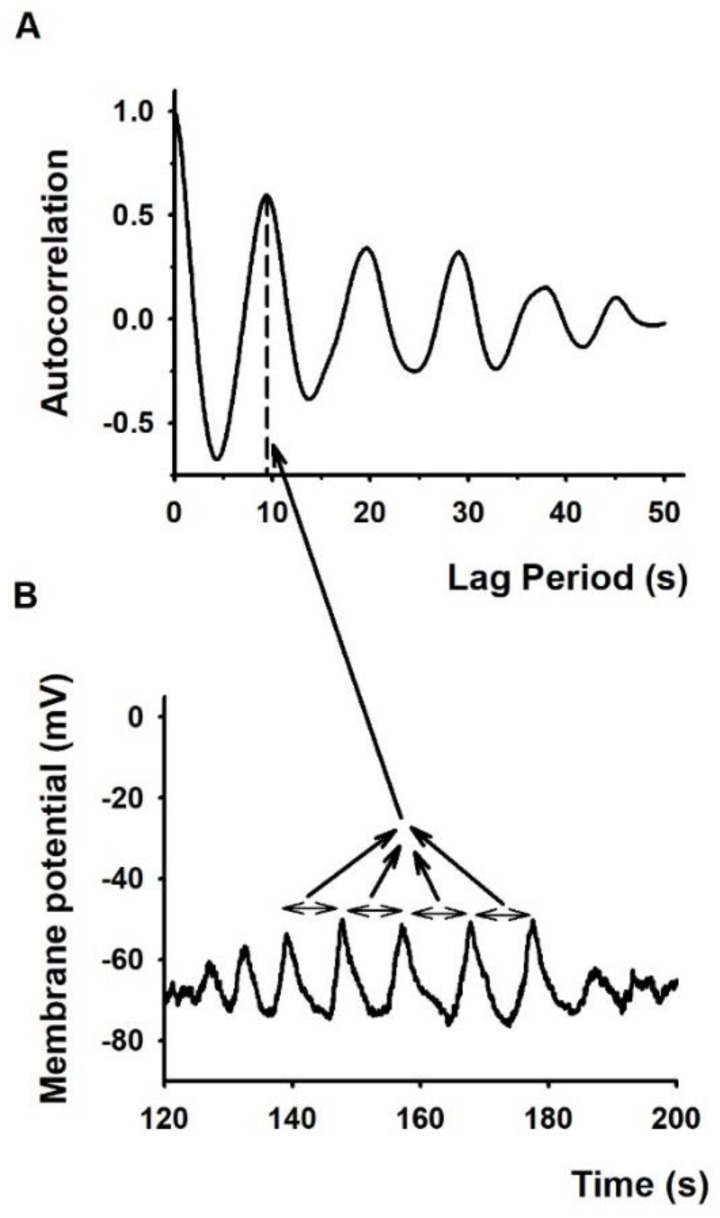
Autocorrelation analysis of membrane potential oscillations. (**A**): Autocorrelation function based on a representative membrane potential oscillation shown in panel (**B**) below recorded from a D10 cell forming an IS. Horizontal arrows in panel B indicate the time interval between peaks. The first peak (at non-zero time) of the autocorrelation function (**A**) indicated by dashed line represents the shortest oscillation time period. This peak at 10 s time lag indicates periodicity change of membrane potential with a frequency of 0.1 Hz.

The amplitudes of oscillations had considerable cell-to-cell variability and changed during the observation time. Figure 3C shows the distribution of oscillation amplitudes recorded from cells engaged in an IS. The figure indicates that the major component of the amplitudes is 13 mV, and there are three more peaks superimposed on this with characteristic amplitudes of 21.5, 30 and 42 mV. The simplest approach to calculate oscillation frequency was based on manually counting the number of peaks within the duration of the oscillation. This method was applicable to records shown in Figure 3A where peaks could easily be identified, whereas the analysis of the MPOs in the presence of ion channel blockers (see below), where MPO components with multiple frequencies were present, required more sophisticated analysis, such as the calculation of the temporal autocorrelation of membrane potential records (Figure 4 and Methods). This analysis yielded an oscillation frequency of 0.075 ± 0.009 s^−1^ (*n* = 13) for MPO records of IS conjugated cells in the absence of ion channel blockers.

### 2.3. Membrane Potential Oscillations Are Sensitive to Potassium Channel Blockers and to Extracellular Calcium

**Figure 5:**
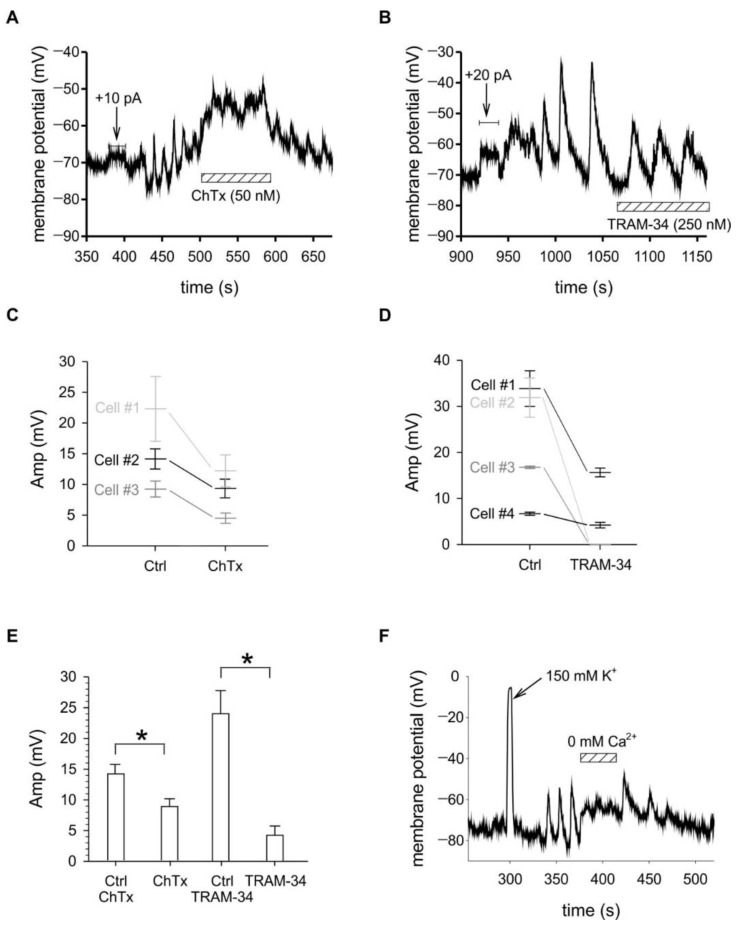
Membrane potential oscillations are sensitive to Kv1.3 and KCa3.1 channel blockers and to extracellular calcium concentration. (**A**,**B**): Representative membrane potential records (current-clamp) of D10 cells conjugated to CH12 cells. MPO was by induced by depolarizing current injections (arrows) with durations indicated by the lengths of the horizontal lines. The hatched horizontal bar indicates perfusion with 50 nM ChTx (**A**) or with 250 nM TRAM-34 (**B**). (**C**): MPO amplitudes of individual cells were averaged (mean ± SEM) in control extracellular solution (Ctrl) and following the perfusion with 50 nM ChTx (ChTx). (**D**): MPO amplitudes of individual cells were averaged (mean ± SEM) in control extracellular solution (Ctrl) and following or 250 nM TRAM-34 (TRAM-34). Oblique lines connect the corresponding average amplitudes in Ctrl and in the presence of the blockers. (**E**): Effect of ChTx and TRAM-34 on the MPO amplitudes. The MPO amplitudes (Amp) were pooled before (Ctrl ChTx) and after 50 nM ChTx (ChTX) or before (Ctrl TRAM-34) and after 250 nM TRAM-34 (TRAM-34) and averaged (mean ± SEM). Asterisks indicate significant differences (* *p* < 0.05). (**F**): Membrane potential record (current-clamp) of a D10 cell conjugated to a CH12 cell. MPO was by induced by perfusing the recording chamber using 150 mM K^+^ extracellular solution (arrow). The hatched horizontal bar represents perfusion with a Ca^2+^-free extracellular solution (0 mM Ca^2+^).

**Figure A2:**
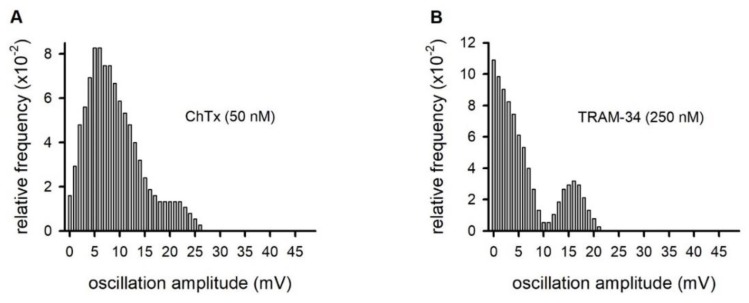
The distribution of MPO amplitudes after the application of ChTx or TRAM-34. Data were extracted from experiments in Figure 5 (**A**) and (**B**). See details there. Relative frequency histograms were constructed as described in the Materials and Methods.

We hypothesized that MPOs result from the alternating activity of depolarizing inward Ca^2+^ and repolarizing outward K^+^ currents, thus interfering with any of the currents is expected to have an impact on their properties. We applied either charybdotoxin [37,38], a potent blocker of both Kv1.3 and KCa3.1 channels or TRAM-34 [33], a selective small-molecule blocker of KCa3.1, in concentrations that cause nearly complete inhibition of the relevant channels. Both ion channel blockers had drastic effects on the membrane potential oscillations (Figure 5). 50 nM charybdotoxin depolarized the membrane potential by 25.3 ± 5.6 mV (*n* = 3) and decreased the amplitude of the MPOs significantly (*p* = 0.013; paired t-test) (Figure 5A,C,E and Figure A2 panel A) to 56 ± 5.1% (*n* = 3) of the control value on average in a reversible manner.

Even though the membrane potential kept fluctuating in the presence of ChTx, the periodicity was severely disturbed precluding accurate frequency determination. TRAM-34 (250 nM) neither depolarized the resting membrane potential nor influenced the frequency of the oscillations but reduced the amplitude of the MPO (Figure 5B,D,E and Figure A2 panel B) to 27 ± 16% (*n* = 4) of the control value on average (*p* = 0.020; paired t-test), moreover, in some cases it completely abolished the MPO. Removal of Ca^2+^ from the extracellular solution also completely obliterated the oscillations confirming the role of Ca^2+^ influx in the phenomenon (Figure 5F).

### 2.4. Oscillation of the Intracellular Calcium Concentration in Conjugated and in Lone T Cells

**Figure 6:**
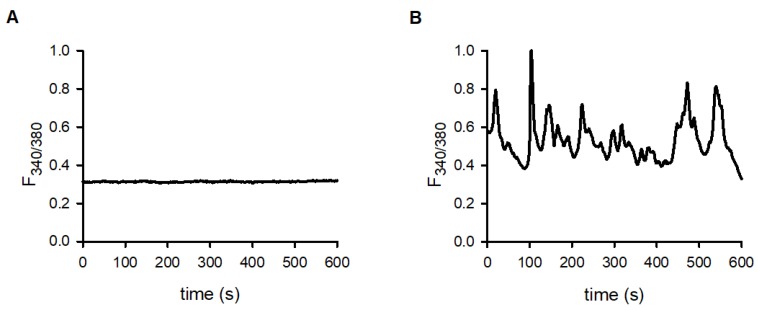
Intracellular calcium concentration in lone and IS conjugated D10 cells. IS conjugated cells were selected based on the PKCθ-GFP recruitment into the IS in conjugated cells (see Figure 2E). Fura-2 florescence was measured at 510 nm upon excitation at 340 nm or 380 nm. Increase in the F_340/380_ ratio reports on the increase in the cytosolic free Ca^2+^ concentration (**A**): F_340/380_ record in a D10 cell not forming an IS. (**B**): F_340/380_ record in a D10 cell engaged in an IS with a CH12 cell.

As early studies suggested the importance of oscillations of the intracellular Ca^2+^ concentration in T cell activation, and MPOs are likely to involve periodic transmembrane Ca^2+^ fluxes, we recorded the changes of the intracellular calcium concentration of D10 cells not conjugated (lone) or conjugated with specific antigen presenting CH12 cells using the fluorescence dye Fura2-AM in ratiometric configuration. In three quarters of the D10 cells engaged in an IS with CH12 B cells (13 out of 17 cells, 76%) we detected spontaneous intracellular calcium oscillations (ICaO) (Figure 6B). In contrast, a much smaller fraction (6 out of 20 cells, 30%) of lone cells produced such oscillations under similar conditions, but most of the cells produced absolutely no ICaO at all (Figure 6A). Similarly to MPOs, the ratio of the occurrence of calcium oscillations was significantly higher in conjugated cells than in lone cells indicating that the presence of ICaO is not independent from the immunological synapse formation (Fisher’s exact test, *p* = 0.0081). The frequency of oscillations in cells forming an IS was 0.036 ± 0.004 Hz (*n* = 13).

## 3. Discussion

Soon after the discovery and characterization of the Kv1.3 channel in T cells, its critical role in T cell activation and proliferation via the modulation of the membrane potential was recognized. Although at different numbers, Kv1.3 is present in T cells of all differentiation and activation states and significantly contributes to the execution of the activation pathway in each type. On the other hand, the expression level of KCa3.1 varies over a wider range among the different T cell subtypes, and therefore has variable influence on the activation process [39]. In our murine T cell model we have shown that KCa3.1 is expressed at levels comparable to Kv1.3 and therefore, when activated by the rise in intracellular Ca^2+^ concentration, it becomes an active participant in the modulation of the membrane potential and Ca^2+^ signaling.

The formation of an immunological synapse was found to be critical for the proper course of signaling pathways leading to T cell activation, including the Ca^2+^ signal. The D10-CH12 model system used in our experiments has been utilized previously to study molecular events of IS formation [34,40]. Following antigen presentation, Kv1.3 channels already present in the membrane were shown to accumulate in the IS [14] and influence Ca^2+^ signaling. When channel redistribution was inhibited by antibody crosslinking, the amplitude of the Ca^2+^ signal was significantly elevated [41]. This is in harmony with the finding that Systemic lupus erythematosus (SLE) T cells show an augmented Ca^2+^ signal that is associated with altered Kv1.3 distribution and faster clearance from the IS compared to healthy T cells [42]. Thus, not only the appropriate number of ion channels, but also their spatial distribution may be a key determinant of the precise shaping of the Ca^2+^ signal.

Earlier studies have described membrane potential fluctuations in T cells, some of them with characteristics of periodic oscillations, as well as oscillations of the Ca^2+^ level, and their connection was also investigated [5,6,7,8,9]. In accordance with these reports we have acquired recordings, in which both the membrane potential or the Ca^2+^ concentration showed high amplitude periodic changes, however, some records lacked these significant fluctuations. By comparing the relative frequency of MPOs and ICaOs in lone cells and those engaged in an IS, we have found that both types of oscillations occurred significantly more frequently in cells engaged in IS. Our conclusion is based on a relatively small number of observations due to the inherent limitation of the single-cell techniques used. For example, to record MPOs, cell conjugates had to be perfectly oriented in the recording chamber to allow detection of PKCΘ-GPF translocation, as an indicator of IS formation, and simultaneously allow the access of the recording patch-clamp pipette to the D10 cell in the conjugate. Although our experimental setup did not allow simultaneous recording of both parameters (MPO and ICaO) on the same cells, the similar relative frequencies of the oscillations in the IS-forming versus lone cells suggests a correlation between them. The average frequencies of MPOs and ICaOs did not match exactly, however, they were in a comparable range, which may indicate a link between the two phenomena. Considering the strong mutual causal connection between transmembrane ion fluxes and membrane potential changes this similarity is not surprising. The lack of a perfect overlap may originate from the experimental limitation mentioned above, i.e., the lack of simultaneous membrane potential and Ca^2+^ measurements on the same cells.

The minimum requirement for alternating membrane potential changes is the presence of ion transporters producing depolarizing and repolarizing currents whose activity is linked to each other creating feedback loops. Such scenario was described for striatal low-threshold spiking interneurons, in which the interplay of two types of voltage-gated Ca^2+^ channels and a calcium-activated chloride channel generated MPOs [43]. In T cells, fluctuations were attributed to the two K^+^ channels described above and the then yet unidentified Ca^2+^ influx pathway, which is now known to be via the CRAC channels [8]. Here we tested the role of these channels in producing MPOs by the application of channel inhibitors and the removal of extracellular Ca^2+^. In this model the current required for depolarization and the subsequent activation of the K^+^ channels is supplied by Ca^2+^ influx through CRAC channels. The requirement for the Ca^2+^ current was confirmed by the complete elimination of MPOs upon the removal of extracellular Ca^2+^. Upon removal of extracellular Ca^2+^ the membrane potential stabilized at its instantaneous value, as seen in Figure 5F., which may be interpreted as the lack of feedback interactions between the channels in the absence of Ca^2+^. Reapplication of Ca^2+^ caused an initial depolarization, as expected, then partially restored oscillations repolarized the membrane as KCa3.1 channels were reactivated. The two K^+^ channels provide the repolarizing current in this model and as expected, their simultaneous inhibition caused significant depolarization and suppression of the MPOs (Figure 5A). Since IS development is a dynamic process [44], which started at the co-centrifugation of the cells, the variable time periods elapsed to the achievement of the whole-cell patch-clamp configurations may have caught different stages of the initial depolarizing Ca^2+^ influx or may even have missed it completely. This may explain why most cells required depolarization either by current injection or high external K^+^ to initiate MPOs, while in a few cells it was spontaneous.

Our most important finding is that the likelihood of both high amplitude MPOs and ICaOs is significantly increased in cells that form an immunological synapse with an antigen presenting cell. Although the membrane potential and intracellular ion concentrations are generally thought of as global properties of a cell, controlled by multiple evenly distributed ion flux pathways, special membrane potential responses require uneven distribution of ion channels producing significant spatial fluctuations in ion concentrations. For example, in neurons, action potential generation and propagation rely on high densities of ion channels at the axon hillock and the nodes of Ranvier interspersed by areas of low channel densities or lacking channels completely.

Furthermore, the clustering of different channel types into multi-channel complexes allows for very efficient and fast interaction and mutual modulation of channel function. This is well exemplified by the co-clustering of the voltage- and Ca^2+^-activated BK potassium channel with several types of voltage-gated Ca^2+^ channels. The membrane potential activation threshold of BK channels depends on the intracellular Ca^2+^ concentration and was found to show strong correlation with the activation threshold voltage of the associated calcium channels [45]. Although the cytosol has strong Ca^2+^ buffering capacity, it has been shown that a single open Ca^2+^ channel can raise the local Ca^2+^ concentration to the high μM range within a few tens of nanometers around the channel for short periods [46,47]. This way Ca^2+^ influx through a Ca^2+^ channel associated with a BK channel can be sufficient to significantly shift the voltage-dependence of BK and activate the channel in the complex. Recently, the significance of channel complexes and Ca^2+^ microdomains during early T cell activation was also demonstrated using super-resolution microscopy [48].

The formation of such channel complexes may be spontaneous but may also be induced by signaling events, such as binding of a mitogen or the formation of an IS. The membrane potential fluctuations attributed to the close interplay of Kv1.3, KCa3.1 and CRAC channels was described in T cells activated by phytohemagglutinin (PHA), which is known to cause cap formation and membrane protein aggregation so it is likely to rearrange ion channel distributions and lead to complex formations [8]. However, even this scenario does not completely mimic the conditions in an IS, where special effects linked to ion channels must be considered. Localized intra- and extracellular ion concentrations may vastly differ from physiological conditions, which can modify the gating of the channels and modulate their contributions to membrane potential control. For example, K^+^ accumulation in the synaptic cleft can slow the inactivation, increase the conductance of Kv1.3 channels and cause depolarization with an ensuing increase in opening probability [49] causing an overall increase in the K^+^ permeability of the membrane facing the cleft. A high density of CRAC channels in the IS can lead to local intracellular Ca^2+^ spikes activating KCa3.1 channels and downstream Ca^2+^-dependent pathways while also causing inactivation of CRAC channels [50,51], and the depletion of Ca^2+^ from the cleft [10]. The decrease in transmembrane ionic gradients due to external K^+^ accumulation and Ca^2+^ depletion may shift the reversal potential of these ions toward zero making the channels in the IS become less effective at controlling the membrane potential, while the channels outside the IS would be activated with a lag due to the time requirement of the signal propagation, introducing a delay in the feedback mechanisms. These slower responses may allow larger deviations of the membrane potential before the counterbalancing process reverses the change, allowing the development of large amplitude oscillations.

To explore the role of K^+^ channels in the MPOs, we applied inhibitors, namely TRAM-34, a specific blocker of KCa3.1, and ChTx, which blocks both Kv1.3 and KCa3.1. As seen on the amplitude histograms the highest amplitude oscillations were abolished by both blockers (Figure 3C and Figure A2). In some cells, the inhibition of KCa3.1 channels only preserved the frequency of oscillations, but reduced their amplitudes, suggesting that under special circumstances Kv1.3 channels may be sufficient to generate MPOs, but with a lower efficiency. In other cells KCa3.1 inhibition caused complete elimination of the MPOs. The variability in channel numbers and distributions may explain the different MPO amplitudes observed in different cells, as well as the varied MPO responses to TRAM-34. Our whole-cell current recordings indicated a wide range of current amplitudes, greater than 6-fold range in KCa3.1 and over 10-fold range in Kv1.3 currents, implying that the ratio of the two channels on individual cells may greatly vary, leading to different sensitivities to TRAM-34. In addition, the kinetics of channel translocation into and out of the IS are different for the channels. Kv1.3 and CRAC channels were shown to localize at the leading edge of migrating T cells, whereas KCa3.1 localized in the trailing part [52], which suggests that their relocation to the forming IS may require different time periods. Moreover, in about half of the observed cells KCa3.1 was reported to reside in the IS for 14–30 min, whereas in the other half this duration was only 7 min [15]. Thus, the time elapsed between the initiation of IS formation and the patch-clamp recording may have influenced channel distributions and consequently MPO amplitudes and the response to TRAM-34.

By inhibiting both K^+^ channels, ChTx almost completely blocked the repolarizing currents and accordingly caused strong depolarization and suppression of the MPOs. The fact that some low amplitude MP fluctuations still persisted in the presence of ChTx may be explained by the obstructed access of the toxin to channels within the enclosed space of the IS. With the diffusion of even small molecules being limited [10,29], the large 4 kDa toxin is unlikely to reach its concentration required for full block of the channels residing in the synaptic cleft. Such an effect was demonstrated by the decreased binding rate of a K^+^ channel blocking toxin to Kv1.3 as a consequence of spatial hindrance by gold nanobeads attached to nearby receptors [53]. Thus, the lower toxin concentration in the cleft may allow a fraction of the channels in the IS facing reduced ionic gradients to modulate the MP, but not to generate high amplitude oscillations.

Although all proposed elements of the MPOs are present even in lone cells, without their special arrangement, generation of MPOs and ICaOs is much less likely. We therefore propose that not only the presence of the channels, but also their spatial distribution and their relative numbers and locations are critical for producing high amplitude and long lasting MPOs and concomitant ICaOs, which provides the Ca^2+^ signal required for efficient T cell activation. Thus, a special role of the IS is to create this distinct arrangement of channels and environment to enhance the generation of these oscillations.

## 4. Materials and Methods

### 4.1. Cells

The derivative of the cloned murine T cell line D10 (D10.G4.1) is specific for the egg-white protein conalbumin and for the MHC protein IA^k^ [34,40] and expresses GFP-tagged PKCΘ [34]. Murine B cell lymphoma cells (CH12.LX IA^k^, referred to as CH12) were used as antigen presenting cells (both cell lines were a gift from Abraham Kupfer, The Johns Hopkins University, Baltimore, MD, USA). The production and culturing of the cells was described elsewhere [34,40]. Antigen pulsing of the APCs and formation of cell conjugates were performed according to Monks et al. [40].

### 4.2. Electrophysiology

The patch-clamp technique in current-clamp mode was used to measure the membrane potential (Multiclamp 700B amplifier Molecular Devices, San Jose, CA, USA). The normal bath solution contained (in mM): 145 NaCl, 5 KCl, 1 MgCl_2_, 2.5 CaCl_2_, 5.5 glucose, 10 HEPES (pH 7.35). The Ca^2+^-free extracellular solution was based on the normal extracellular one except CaCl_2_ was omitted and 1 mM EGTA was added. The 150 mM K^+^ extracellular solution contained (in mM) 150 KCl, 1 MgCl_2_, 2.5 CaCl_2_, 5.5 glucose, 10 HEPES (pH 7.35). The pipette filling solution contained (in mM): 150 KCl, 2 MgCl_2_, 8.7 CaCl_2_, 5 HEPES, 10 EGTA (pH 7.2). Whole-cell Kv1.3 and KCa3.1 currents were measured in voltage-clamped cells [54] using pipette solutions containing (in mM) 140 KF, 2 MgCl_2_, 1CaCl_2_, 10 HEPES, 11 EGTA (pH 7.2), and 150 K-aspartate, 5 HEPES, 10 EGTA, 8.7 CaCl_2_, 2 MgCl_2_ (pH 7.2), respectively. Cells were identified for recording by using a TE2000 fluorescence microscope (Nikon, Auroscience, Budapest, Hungary).

### 4.3. Intracellular Calcium Measurements

Intracellular ratiometric calcium measurements were made with microscopic imaging technique. D10 cells were loaded with 3 µM Fura-2AM in serum free- buffer for 20 min at 37 °C prior to immunological synapse formation. Subsequently, IS-forming D10 and CH12 were attached to 35 mm glass bottom dishes for 40 min at 37 °C in FBS containing cell culture media. After attachment, image stacks with 340 nm and 380 nm excitation wavelength were recorded every 2 s. Images in bright field and GFP channels were also recorded for cell and IS identification. Images were acquired with a VisiFluor Imaging System (Visitron Systems GmbH, Puchheim, Germany) consisting of a Nikon Eclipse Ts2R-FL microscope, VisiChrome Polychromatic illumination-system, pco.edge 4.2 m HQ camera and VisiView 4.0 image acquisition software. Images were evaluated using the VisiView 4.0 software (Visitron Systems GmbH, Puchheim, Germany) and standard spreadsheets.

### 4.4. Labeling of Kv1.3 Channels

Endogenous Kv1.3 channels of D10 cells, which were conjugated to CH12 cells (20 min incubation at 37 °C) and adhered to poly-L-lysine coated coverslips, were labeled on ice indirectly. The primary antibody was raised against the extracellular loop between S1 and S2 segments of the channel protein (amino acids 211–224: KDYPASTSQDSFEA(C)) (cat #: APC-101, 1:200 dilution, Alomone Labs, Jerusalem, Israel), the secondary antibody was a Cy3 conjugated donkey anti-rabbit IgG (1:500 dilution, The Jackson Laboratory, Sacramento, CA USA). In the end cells were fixed using 4% of formaldehyde and Mowiol (Calbiochem Sigma-Aldrich, St. Louis, MO, USA) was added to the samples before mounting onto slides to prevent photobleaching.

### 4.5. Confocal Microscopy

Images were recorded using a FV1000 confocal microscope (60× UPLSAPO oil immersion objective, NA:1.35, Olympus, Hamburg, Germany) with 1 μm optical slice thickness.

### 4.6. Determination of the presence of MPO

**Figure A3:**
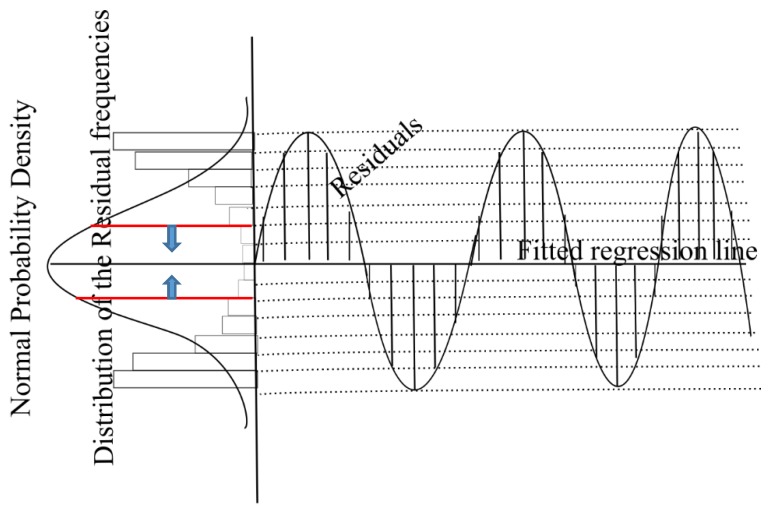
Determination of the presence of MPO.

For objective identification of oscillations we used a mathematical analysis of the recorded membrane potential traces using a methodology adapted and extended from Vizvari and Bacsi [55] (Figure A3). The signal is first normalized by subtracting the minimum and dividing by the range, thereby being transformed into the [0,1] interval. To account for baseline drifts a trend is fitted to the normalized signal (a polynomial of a degree of 3) and the residuals are determined by subtracting the trend from the normalized signal. Then we sum up the residuals for all the investigated recordings creating a single residual distribution function. We then fit a normal distribution to this single residual distribution and bin the values into [log2(n)] + 1 equal segments according to Sturges’ formula. If the residual values are stable (no oscillation), many of them are in the middle segments located around zero, therefore the middle four segments were taken into consideration as favorable segments. The favorable residual frequency (FRF) is the fraction of residuals falling into the middle four segments and the favorable normal frequency (FNF) is the fraction of the area from the fitted normal distribution of the residuals falling within the middle four segments. The oscillation stability index (OSI) is calculated as the difference of the FRF and the FNF as follows Vizvari and Bacsi [55]: OSI = 2*(FRF − FNF).

The OSI index values should fall between −2 and 2, because both FRF and FNF can take values between 0 and 1. Negative values indicate that the distribution of the residuals significantly deviates from normal and implies oscillation. An OSI of 0 or greater suggests residuals with a distribution close to normal with few deviating values indicating the lack of oscillations. The graphical presentation of the algorithm can be seen on Figure A3. in case of a sinus-like signal. Our development compared to the above presented methodology is that we further divided the middle four segments to 200 parts and we calculated the OSI value by gradually narrowing the border (red lines in Figure A3) of the middle four segments on both sides closer to 0 in 100 steps. In the next step we calculate how many times the OSI values fall below 0. In this way we get a final index (between 0 and 100), 0 means no oscillation, the higher values indicate stronger oscillations. The derived index can be used to compare signal oscillations.

### 4.7. Analysis of the MPO amplitudes

**Figure A4:**
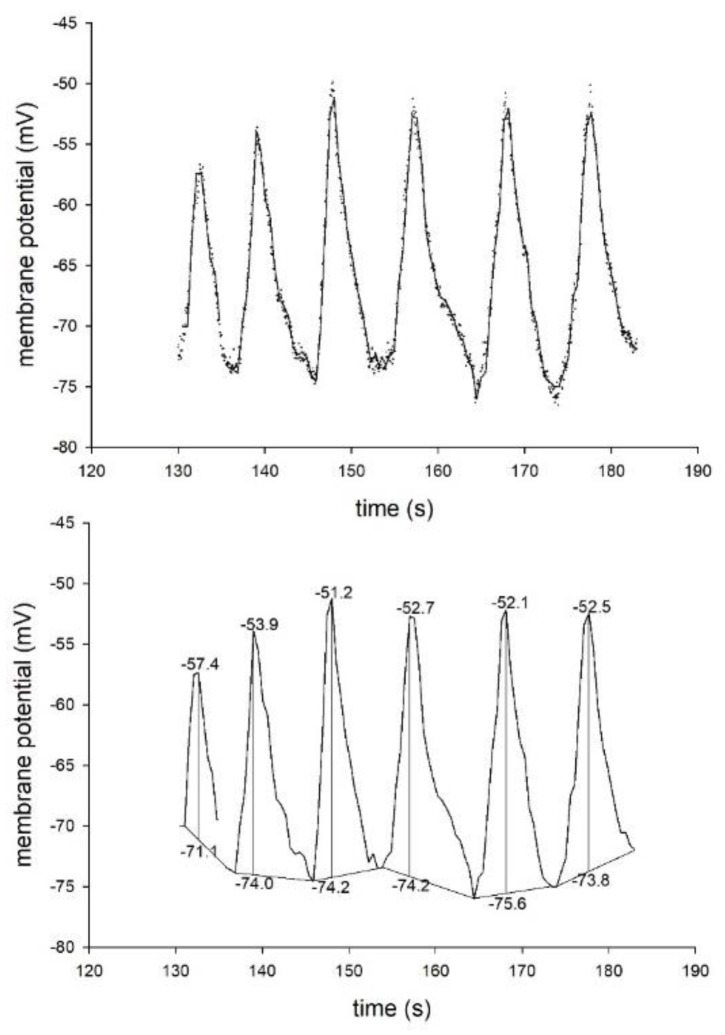
Membrane potential amplitude analysis. Negative peaks were identified in the membrane potential record, connected by straight lines and the positive peaks were measured from these straight lines as base lines (Figure A4). E.g. the first amplitude in Figure A4 illustrating this analysis would be (−71.1) − (−57.4) = −13.7, where −57.4 is the positive peak value and −71.1 is the value where the vertical line (which starts from the positive peak) crosses the base line.

Negative peaks were identified in the membrane potential record, connected by straight lines and the positive peaks were measured from these straight lines as base lines (Figure A4). E.g. the first amplitude in Figure A4 illustrating this analysis would be (−71.1) − (−57.4) = −13.7, where −57.4 is the positive peak value and −71.1 is the value where the vertical line (which starts from the positive peak) crosses the base line. The probability density function of the amplitudes was assessed by creating a smooth histogram. The bin width was 5 mV, and bins were shifted in 4 steps by 1 mV to create a relative frequency value at each mV, where the actual value was assigned to the center of the bin. This procedure resulted in an optimal nonparametric estimator for the probability density function from our data [56].

### 4.8. Statistical Analysis

The SigmaStat software package (Systat Software, San Jose, CA, USA) was used for the statistical analysis (paired t-test, independence). 2 × 2 contingency tables were analyzed using Fisher’s exact test due to the relatively small number of observations and the small number of expected occurrences in some cells. In all statistical tests *p* < 0.05 was considered as significant.

## Abbreviations

TCR	T-cell receptor
CD3	Cluster of differentiation 3
CRAC	Ca^2+^-release activated Ca^2+^
MP	Membrane potential
MPO	Membrane potential oscillation
ICaO	Intracellular calcium oscillation
Kv	Voltage gated K^+^ ion channels
K_Ca_	Calcium activated K+ ion channels
STIM1	Stromal interaction molecule 1
ER	Endoplasmic reticulum
IS	Immunological synapse
APC	Antigen-presenting cell
ChTx	Charybdotoxin
MgTx	Margatoxin
GFP	Green fluorescein protein

## Appendix A

Figure A3 Graphical representation of the algorithm for the objective determination of the presence of MPO in case of a sinus-like signal. The sinus-like oscillating signal is shown on the right hand side. The signal is first normalized by subtracting the minimum and divided by the range, thereby being transformed into the [0,1] interval. To account for baseline drifts a trend is fitted to the normalized signal (third degree polynomial, in general); for this sinus-like signal, the fitted trend line is indicated by a horizontal solid straight line. The residuals are determined by subtracting the trend from the normalized signal. Frequency distribution of the residuals calculated from this model signal is shown on the left hand side of the figure. The Normal Probability Density function on the left hand side is fitted to the residuals for all the investigated recordings creating a single residual distribution function. We then bin the values into [log2(n)] + 1 equal segments according to Sturges’ formula. The red lines indicate the four middle segments of the frequency histogram used for the calculation of the OSI (see details in the Materials and Methods).
